# 

**DOI:** 10.1177/2041669515614148

**Published:** 2015-12-31

Serial effects in perception have been studied since the dawn of psychophysics. Color aftereffects greatly advanced the understanding of color vision in the 19th century, and motion aftereffects have intrigued perceptual scientists for centuries. Recent discoveries in visual attention and psychophysics have intensifed interest in such effects. The current consensus is that they are not curiosities but serve an important function and can be critical for understanding perception.

In December 2015, a small group of vision scientists gathered for a 2-day workshop in Pisa to discuss these issues. The meeting (Serial Effects in Perception: Prediction, Priming, and Adaptation, December 11–12, 2014: www.pisavisionlab.org/sfx2014) brought together experts in varied types of serial effects: perceptual continuity, serial effects, priming, and adaptation. The abstracts of the meeting follow this introduction.

The aim was to discuss the various findings in an attempt to find common features as well as distinguishing traits. In the first session, on perceptual serial dependencies, David Whitney presented evidence that both lower and higher level perceptual mechanisms utilize assimilative trial to trial information: Current perceptions are biased toward previous ones (Fischer & Whitney, 2014; Liberman, Fischer et al., 2014). Whitney also introduced the concept of a “continuity field”: A zone in space and time where the brain looks for continuities. Although the extent of the continuity field might depend on the particular technique used for stimulus presentation and response, the concept seems useful to describe the benefits of serial effects: Things rarely change rapidly in the world so it might be beneficial to draw upon several snapshots overtime to represent the environment.

Cicchini and Burr tackled a complementary issue. According to their work, a simple formula determines the optimal weight of current and past information: If two stimuli are similar, it is beneficial to give a consistent weight to past information and this reduces noise; however, if two stimuli are markedly different, the weight of the past information should be very small. In this way, one obtains an optimal mechanism that discounts sensory fluctuations, yet is responsive to abrupt changes (Burr & Cicchini, 2014). Such an adaptive filter, akin to a Kalman filter, fits well with several findings on serial dependencies and this strategy may be incorporated in biological systems (Cicchini, Anobile, et al., 2014). Cicchini et al. showed how this model predicts nonlinearities in number-to-line mappings, previously thought to reflect logarithmic encoding (Dehaene, Izard, et al., 2008), and also provided new evidence that positive serial dependencies can occur at a strictly perceptual level even with diverse intervening responses.

The aim of the second session was to provide a picture of the current understanding of the brain as a mechanism for hypothesis-generation (aka prediction). Lars Muckli presented recent work showing that the brain automatically perceives stimulus change and extrapolates possible future stimulus features. Muckli used two-frame apparent motion finding that the brain is capable of both filling in missing frames as well as extrapolating the object trajectory (Muckli, Kohler, et al., 2005; Weigelt, Kourtzi, et al., 2007). Interestingly, his research also reveals considerable crosstalk between areas dedicated to motion perception and areas involved in motion prediction providing a strong argument that prediction is a critical brain function occurring even at the first stages of processing (Alink, Schwiedrzik, et al., 2010; Kaas, Weigelt, et al., 2010; Muckli, Kohler, et al., 2005; Weigelt, Kourtzi, et al., 2007; Wibral, Bledowski, et al., 2009).

Floris de Lange followed up on this, discussing neurophysiological correlates of predictive coding. Predictive coding proposes that perception is the result of two separate processes that complement one another: predictions based on the current state of environment and mismatches between predictions and sensory signals (Friston, 2010; Rao & Ballard, 1999). Spatial and temporal context are known to play a major role in visual perception (Oliva & Torralba, 2007), but the precise mechanisms by which context and expectations modify the neural codes are still poorly understood. de Lange demonstrated that knowing the features of a stimulus in advance changes neuronal responses even as early as in V1 (Kok & de Lange, 2014; Kok, Brouwer, et al., 2013). Interestingly, repeated presentation of a stimulus produces weaker responses, consistent with well-known findings of neural repetition suppression with repeated search displays (Bichot & Schall, 1999; Kristjansson, Vuilleumier, et al., 2007); yet, the information content after repetition increases, consistent with the idea that serial presentation leads to optimization of perceptual processing.

Aldo Genovesio discussed the effects of stimuli and task on previous trials on prefrontal cortical activity in monkeys. His data are particularly revealing in that even when not explicitly required by task, past trial information is stored in a subset of prefrontal neurons (Genovesio, Brasted, et al., 2005; Genovesio, Brasted, et al., 2006; Genovesio, Tsujimoto, et al., 2006; Genovesio, Tsujimoto, et al., 2012; Tsujimoto, Genovesio, et al., 2012). Genovesio hypothesized that this information is crucial for the monkey’s ability to understand and monitor its own actions and is, therefore, critical for learning and reward (Genovesio & Ferraina, 2014). Overall, this suggests that remembering the task is an automatic process that is nevertheless an active and important part of human cognitive abilities.

The second day of the conference started with a session on adaptation. Adaptation attracts interest not only because it is an intriguing form of plasticity and flexibility of brain circuits but also because it has proven an invaluable tool in psychophysics to watermark visual mechanisms and test their specificity (Thompson & Burr, 2009). There are, nevertheless many intriguing open questions, even paradoxes, regarding adaptation. Is adaptation beneficial? And, if so, why is it often accompanied by reduced neural activity?

In the first talk of the second day, Solomon argued that even seemingly simple brain circuits which exhibit surround inhibition, can, when adapted, display a host of behaviors depending on the specific experimental procedure and question (Solomon & Kohn, 2014). However, Solomon warned about inferring functional principles from neural activity unless all details are taken into account. Also, a clear conclusion from Solomon’s talk was that serial effects may come in many guises even though they obey a few simple principles.

Peter Foldiak discussed decorrelation and Anti-Hebbian adaptation. The main thrust of this work is that if two sensory signals are similar because of mutual correlation (such as when the two signals monitor similar parts of the visual field or when they are corrupted by the same noise source), it may be beneficial to decorrelate them by subtracting one from the other, increasing discriminability among the two signals. On a face value, this classic work may seem at odds with the approach of Kalman filtering which suggests that the benefit lies in averaging rather than subtracting stimuli. However, there are quite a few differences between the approaches; for instance, in Foldiak’s work the goal of the system is to discriminate the various signals, whereas in Kalman filtering the goal is to have representations close to veridical. Also, another crucial issue is the nature of noise. In the standard version of the Kalman filter, noise is assumed independent, whereas in the decorrelation framework, it is assumed that the same noisy information sneaks into both signals (de Gardelle & Summerfield, 2011). Indeed, a final framework for assessing which mechanism is optimal needs to take into account several details and the answer, likely will vary accordingly.

Peter Thompson closed the session, describing an example of cross-modal adaptation, which he cleverly used as a tool to demonstrate multimodal representations in the brain. Such cross-modal adaptation effects are extremely interesting, as they have notable implications for understanding the mechanisms involved in adaptation and the level at which they occur.

One of the themes of the workshop was to bridge gaps between disciplines. While the interest in sequential dependencies in basic vision science is in many cases recent, a large literature spanning over 20 years has emerged within the field of visual attention, showing how recent attention deployments strongly influence future ones.

Typically, attentional priming shows how response times are speeded (Maljkovic & Nakayama, 1994) or accuracy increased (Asgeirsson, Kristjansson, et al., 2014) if attentional priorities remain constant between trials. Priming can have a dominating influence on attentional functioning (for review, Kristjansson & Campana, 2010; Kristjansson, Heimisson, et al., 2013), but while priming is often merely thought of as a facilitation of reaction times, having little relevance to perception, Arni Kristjansson was able to show that under the appropriate circumstances priming affects attentional choice (Brascamp, Pels, et al., 2011), overcomes masking (Asgeirsson & Kristjansson, 2011; Sigurdardottir, Kristjansson, et al., 2008), and releases items from crowding (Kristjansson, Heimisson, et al., 2013), which has strong implications for perception.

Gianluca Campana attempted to pin down the neural substrates of priming and to what extent areas involved in motion extrapolation share resources with those for perception. Campana showed that motion extrapolation in adapted regions of the visual field is affected consistently (Battaglini, Campana, et al., 2015). Along the same lines, he showed that if motion perception is primed (by exposure to a moving patch), time to contact estimation is affected. Overall, these results support a strong entwinement between mechanisms for perception, prediction, and extrapolation, which may connect priming to mechanisms of prediction (Kanai & Verstraten, 2005).

Lastly, Mamassian made the case for multiple scales of adaptation in the brain. Chopin and Mamassian (2012) analyzed effects of recent or remote stimuli on the perception of an oriented grating and found that while recent stimuli have a repulsive effect, more distant trials in time exert a positive aftereffect on perception. Mamassian presented evidence that the strongest repulsive aftereffects occur at the beginning of a series of stimuli with similar intensity (McDermott, Chopin, et al., 2015). Interestingly, there were both biases in perceived motion direction (adaptation) in addition to a reduction in discrimination threshold, reflecting perceptual learning. The two may not necessarily occur on the same timescales, however.

The last session of the workshop dealt with how we build averages of the perceptual input and infer the statistics and rules of novel environments or settings.

József Fiser presented evidence that the brain employs Bayesian principles in learning which nicely complemented Maloney’s presentation (see below) and extended the Bayesian algorithms (that take reliability into account) to the purposeful learning of cognitive associations. Second, Fiser examined what determines intertrial dependencies, by manipulating stimulus sequences so that the sequence itself was predictable for the observer. In this way, Fiser demonstrated that under some circumstances observers can incorporate the precise structure of the sequence to perform better. Simultaneously, however, observers display regression toward the mean and short-term intertrial serial effects.

Geoff Boynton presented work where a train of stimuli that varied on several features (size, spatial frequency, position) was presented and observers had to reproduce the average stimulus. While, for several dimensions, a recency effect was seen (the latest stimuli contributing most to the average), positional judgments displayed a primacy effect with the first one or two stimuli dictating the average (Hubert-Wallander and Boynton, 2015). These data present a challenge for the idea that a single unifying concept may account for all serial effects.

Lastly, Larry Maloney investigated the time course of learning a new set of motor rules, what determines learning, and whether the rules can be stored and retrieved as necessary. In his experiment, people reached to a target and estimated their chances of success. Without warning, participants’ elbows were restrained so that reaching movements could only be performed by moving the trunk. Subjects again gained a new appropriate motor behavior. Observers’ behavior could be modeled successfully with Bayesian updating, a strategy which weights information by its novelty and reliability, akin to the Kalman filter invoked by Burr and Cicchini (2014). In essence, it seems that reliability-based updating might occur at different scales of processing in the brain.

Overall, this gathering of researchers from differing subdisciplines was inspiring. Clearly, history effects are ubiquitous in perception, seen in many paradigms that probably reflect a number of differing mechanisms. As for the question of finding a unifying framework for serial effects, there are clear obstacles to that. The diversity of paradigms employed and limited knowledge of several, potentially important variables suggest that the task will be difficult.

Nevertheless, several considerations may indicate that the varied results might become part of a larger, framework that explains how the past is used to predict the present:

1. Natural statistics contain strong serial correlations as the world tends to be stable from moment to moment. So, whether it is a window spanning several seconds as Whitney suggests, or a flexible self-adjusting mechanism as Burr and Cicchini propose, it is highly likely that the brain has developed strategies to exploit such regularities. Indeed, in some perceptual domains such as motion integration or transaccadic perception, the brain displays a remarkable ability to integrate information over large time intervals (Burr & Morrone, 1996; Burr & Santoro, 2001; Cicchini, Binda, et al., 2013; Neri, Morrone, et al., 1998; Panichi, Burr, et al., 2012).
2. Although the human brain is remarkably flexible, we often engage in continuous or sustained tasks that would benefit from fine tuning of responses rather than flexibility (Kristjansson & Campana, 2010). It may be beneficial to keep track of what we have been doing before instead of starting afresh. This feature should not be underestimated as it might be critical in many task of fine skills and might even enable learning itself, as Genovesio suggests (Genovesio & Ferraina, 2014).
3. The brain is capable of decoding long chunks of information overtime, such as scenes or movies. It possesses the ability to elaborate and recognize information spread overtime. In normal perception what are these mechanisms up to? Are they inactive or do they play a strong role and enable predictions?

All this highlights that a common framework for serial dependencies might be feasible. A central governing principle might involve the benefits of taking the past into account while interpreting the present environment. (Busse, Ayaz, et al., 2011; Frund, Wichmann, et al., 2014).

It is easy to envision teleological reasons behind serial effects while science demands empirical demonstrations. The findings presented at the meeting hopefully serve this goal and bring to mind the idea of living organisms living in a 4D world where temporal information and context is as important and informative as spatial context.

What is needed for a firmer grounding for this framework? Clearly, it would be of help to see whether serial dependencies vary consistently with the amount of available information or whether they reflect fixed mechanisms operating on precise timescales. Possibly one of the most urgent tasks is to demonstrate up to which point serial dependencies comply with rules of optimal integration and bring about a real decrease of stimulus uncertainty.

At the same time, it is crucial to understand why priming is sometimes positive and sometimes negative (Kanai & Verstraten, 2005). Possibly, the recent finding that negative priming is retinotopic and positive priming is spatiotopic may help in this (Yoshimoto, Uchida-Ota, et al., 2014). This is an oddity that cannot be dismissed by simply saying that the brain tracks temporal correlations in natural scenes.

The results that were, perhaps, the most surprising were Boynton’s. Why, all other things being equal, do we set our minds with the first stimuli while for other tasks we draw on our recent past. How could the continuity field approach, or even a Bayesian framework, accommodate these results? A possible explanation is that repetition effects operate on varied timescales (see e.g., Brascamp, Pels, & Kristjansson, 2011) and reflect the operation of diverse neural mechanisms, but firmer evidence is needed to clinch such an argument.

If a unitary framework for serial dependencies in vision is to emerge, it will surely take time. Yet, the participants of the workshop were in agreement that such meetings help in constructing bridges between fields of research, which is crucial for any such progress.
