# Supplementary material for: Abstracts
Source: Iperception. 2015 Dec 31;6(6):2041669515614148. doi: 10.1177/2041669515614148 (PMC4975114; doi:10.1177/2041669515614148)
Supplement: Related texts

# Sub article: The Continuity Field: A Mechanism for Perceptual Stability via Serial Dependence

**Authors:** David Whitney, Jason Fischer, Alina Liberman

**Affiliations:** University of California, Berkeley, CA; Massachusetts Institute of Technology; University of California, Berkeley

## Abstract

A critical function of vision is to maintain stable perception of objects so they look the same from moment to moment. This is a challenge because visual input is noisy and discontinuous due to, for example, shadows, motion, occlusion, changes in lighting, noise, and so forth. Although a classic question, the mechanism that links the perception of an object’s identity and properties from moment to moment is unknown. Here, we demonstrate the perceptual constancy field, a novel mechanism of object constancy built on serial dependence. We found that on a given trial, a subject’s perception of the orientation of a grating reflected not only the currently viewed stimulus but also a systematic attraction toward the orientations of the previously viewed stimuli. We found the same serial dependence in the perception of facial expression. This perceptual attraction extended over several trials and seconds and displayed clear tuning to the difference (in orientation or facial expression) between the sequential stimuli. Furthermore, serial dependence in object perception was spatially specific and selective to the attended object within a scene. Several control experiments showed that the perceptual serial dependence that we report cannot be explained by effects of priming, known hysteresis effects, visual short-term memory, or expectation. Instead, there is a systematic influence of recent visual experiences on perception at any given moment: Visual percepts, even of unambiguous stimuli, are attracted toward what was previously seen. Our results reveal a novel mechanism—the Continuity Field—that helps maintain stable object and scene representations in the face of a dynamic and noisy environment.

# Sub article: 1.2 Serial Dependencies Cause Logarithmic-Like Compression of the Numberline

**Authors:** Guido M. Cicchini, Giovanni Anobile, David Burr

**Affiliations:** CNR Institute of Neuroscience, Italy; University of Florence, Italy; CNR Institute of Neuroscience, Italy and University of Florence, Italy

## Abstract

Mapping of number onto space is fundamental to measurement and mathematics. However, numberline mapping of young children, unschooled adults, and adults under attentional load or making speeded responses shows strong compressive nonlinearities, thought to reflect intrinsic logarithmic mechanisms, later “linearized” by education. Here, we advance and test an alternative explanation: that the nonlinearity results from adaptive mechanisms incorporating the statistics of recent stimuli. This theory predicts strong serial dependencies, whereas a static logarithmic nonlinearity predicts trial-wise independence. We found strong and highly significant correlations between numberline mapping of the current trial and the magnitude of the previous trial, in both adults and school children. The dependencies were particularly strong at the beginning of pointing trajectories (Dotan & Dehaene, 2014). The current response was influenced by up to 20% by the previous trial value is sufficient to account quantitatively for the compressive shape of the numberline. A Bayesian-like model shows that the dynamic serial dependency leads to a reduction of reproduction error and hence improvement in overall accuracy.

## References

[bibr46-2041669515614148] DotanD.DehaeneS. (2014) How do we convert a number into a finger trajectory? Cognition 129: 512–529.2404183710.1016/j.cognition.2013.07.007

# Sub article: 2.1 Cortical Predictions During Motion Illusions

**Authors:** Lars Muckli

**Affiliations:** University of Glasgow, UK

## Abstract

We have used a variety of motion illusions to investigate serial dependency processing in our cortical visual system measured with functional brain imaging. Using two-dimensional long-range apparent motion, three dimensional rotational apparent motion, and complex scene motion, we found that different stages of cortical processing fill-in intermediate object positions and extrapolate to upcoming predicted visual scenes (Alink, Schwiedrzik, Kohler et al., 2010; Maus, Weigelt, Nijhawan, et al., 2010; Vetter, Edwards, & Muckli, 2012; Vetter, Grosbras, & Muckli, 2013; Weigelt, Kourtzi, Kohler, et al., 2007). The cortical visual system extrapolates intermediate stimulation positions and broadcasts this expectation across different hierarchical processing stages. Motion extrapolations rely only on conscious illusion perception when the temporal integration becomes longer (Vetter et al., 2014). Thus, serial dependency is tracked by the visual system with and without conscious perception of motion. The findings illustrate how the visual system works in synergy across different processing stages to anticipate upcoming visual stimulation.

## References

[bibr47-2041669515614148] Alink, A., Schwiedrzik, C. M., Kohler, A., Singer, W., & Muckli, L. (2010). Stimulus predictability reduces responses in primary visual cortex. *Journal of Neuroscience, 30*, 2960–2966.10.1523/JNEUROSCI.3730-10.2010PMC663395020181593

[bibr48-2041669515614148] MausG. W.WeigeltS.NijhawanR.MuckliL. (2010) Does area V3A predict positions of moving objects? Frontiers in Psychology 1: 186.2189782410.3389/fpsyg.2010.00186PMC3158432

[bibr49-2041669515614148] VetterP.EdwardsG.MuckliL. (2012) Transfer of predictive signals across saccades. Frontiers in Psychology 8: 176.2270110710.3389/fpsyg.2012.00176PMC3370329

[bibr50-2041669515614148] Vetter, P., Grosbras, M. H., & Muckli, L. (2013). TMS over V5 disrupts motion prediction. *Cerebral Cortex*.10.1093/cercor/bht297PMC438000224152544

[bibr51-2041669515614148] VetterP.SandersL. L. O.MuckliL. (2014) Dissociation of prediction from conscious perception. Perception 43: 1107–1113.2550968710.1068/p7766

[bibr52-2041669515614148] WeigeltS.KourtziZ.KohlerA.SingerW.MuckliL. (2007) The cortical representation of objects rotating in depth. Journal of Neuroscience 27: 3864–3874.1740925110.1523/JNEUROSCI.0340-07.2007PMC6672396

# Sub article: 2.2 How Do Prior Expectations Change Sensory Processing?

**Authors:** Floris de Lange

**Affiliations:** Radboud University, Nijmegen, Netherlands

## Abstract

Although there is ample behavioral evidence for the predictive nature of perception, the incorporation of prior expectations in the neural computations underlying perception is still poorly understood.

In my lecture, I will review recent work on how prior expectations about the sensory world change the neural computations that give rise to perception. I will highlight recent neuroimaging data (Magnetoencephalography [MEG] and functional Magnetic Resonance Imaging [fMRI]) and methods (multivariate classification techniques) that show how stimulus expectations modify the sensory response. I will also contrast the effects of prior expectation with the modulatory effects of selective attention and adaptation.

In the spirit of the workshop, I will also focus on new data and ideas that we are currently testing with respect to serial effects in perception.

# Sub article: 2.3 Influence of Trial History on Prefrontal Cortex Activity in the Macaque Monkeys

**Authors:** Aldo Genovesio

**Affiliations:** University of Rome “La Sapienza”, Italy

## Abstract

Monitoring the trial history in terms of the animal’s previous choices can be important not only when required by the task but also when irrelevant to the task. We started to study the dorsolateral prefrontal cortex representation of the past spatial goals adopting a strategy task in which maintaining previous goal information across trials was a task requirement (Genovesio, Brasted, & Wise, 2006). This task required the monkeys to stay or shift from the previous response when the stimulus presented in a given trial was either the same or different, respectively. We identified separate populations of neurons encoding past and future goals. One function underneath this separation could be to allow an efficient output motoring function directed to establish whether a previously planned goal was accomplished or still pending. This result is in contrast with the absence of trial history modulation of other prefrontal areas such as the orbitofrontal cortex and the frontal pole cortex (Tsujimoto, Genovesio, & Wise, 2012). In a more recent study (Genovesio, Tsujimoto, Navarra, et al., 2014), we tested whether the prefrontal cortex’s neurons were modulated by the trial history even when not relevant to the task. We studied the activity of dorsolateral and periarcuate neurons in a distance discrimination task in which the monkeys were required to discriminate the distance from screen center of two stimuli, one blue circle and one red square, presented sequentially, by choosing the stimulus farther from screen center when the two stimuli appeared together to the right and to the left of center. We found that neurons represented the previous goal location and its color but not others equally not relevant information such as the distance and the color of the stimuli presented in the previous trials (Genovesio et al., 2014). This result indicates that the monitoring function of the dorsolateral prefrontal cortex although goes beyond task relevance remains, however, restricted to goal information. Previous goal information is likely to be maintained in memory for many purposes such as exploring new alternatives in the exploration or exploitation tradeoff or for finding new problem-solving strategies (Genovesio & Ferraina, 2014).

## References

[bibr53-2041669515614148] GenovesioA.BrastedP. J.WiseS. P. (2006) Representation of future and previous spatial goals by separate neural populations in prefrontal cortex. Journal of Neuroscience 26: 7305–7316. doi:10.1523/JNEUROSCI.0699-06.2006.1682298810.1523/JNEUROSCI.0699-06.2006PMC1501084

[bibr54-2041669515614148] GenovesioA.FerrainaS. (2014) The influence of recent decisions on future goal selection. Philosophical Transactions of the Royal Society B: Biological Sciences 369: 20130477doi:10.1098/rstb.2013.0477.10.1098/rstb.2013.0477PMC418623025267819

[bibr55-2041669515614148] GenovesioA.TsujimotoS.NavarraG.FalconeR.WiseS. P. (2014) Autonomous encoding of irrelevant goals and outcomes by prefrontal cortex neurons. Journal of Neuroscience 34: 1970–1978. doi:10.1523/JNEUROSCI.3228-13.2014.2447837610.1523/JNEUROSCI.3228-13.2014PMC3905153

[bibr56-2041669515614148] TsujimotoS.GenovesioA.WiseS. P. (2012) Neuronal activity during a cued strategy task: Comparison of dorsolateral, orbital, and polar prefrontal cortex. The Journal of Neuroscience 32: 11017–11031. doi:10.1523/JNEUROSCI.1230-12.2012.2287593510.1523/JNEUROSCI.1230-12.2012PMC3466613

# Sub article: 3.1 Adaptation in the Context of Normalization

**Authors:** Samuel G. Solomon, Adam Kohn

**Affiliations:** University College London, UK; Albert Einstein College of Medicine, NY, USA

## Abstract

How an object is perceived depends on the temporal context in which it is encountered. Sensory signals in the brain also depend on temporal context, a phenomenon often referred to as adaptation. Traditional descriptions of adaptation effects emphasize various forms of response fatigue in single neurons, which grow in strength with exposure to a stimulus. Recent work on vision, and other sensory modalities, has shown that this description has substantial shortcomings. Here, we review our emerging understanding of how adaptation alters the balance between excitatory and suppressive signals, how effects depend on adaptation duration, and how adaptation influences representations that are distributed within and across multiple brain structures. This work points to a sophisticated set of mechanisms for adjusting to recent sensory experience and suggests new avenues for understanding their function.

# Sub article: 3.2 Anti-Hebbian Decorrelation as a Model of Sensory Adaptation

**Authors:** Peter Foldiak

**Affiliations:** University of St. Andrews, Scotland, UK

## Abstract

The anti-Hebbian hypothesis states that the strengthening of inhibition or the weakening of excitation between correlated neurons or channels is a functionally important mechanism for the formation of neural connections necessary for the formation of sensory representations. Sensory adaptation and aftereffects can be seen as a side effect of this general mechanism. I will review a range of relevant neurophysiological, psychophysical, and computational results that have emerged since this hypothesis has been first proposed.

# Sub article: 3.3 Cross-Modal Transfer of the Tilt-Aftereffect From Vision to Touch

**Authors:** Peter Thompson, Dafni Krystallidou

**Affiliations:** University of York, UK

## Abstract

There is increasing evidence that some aftereffects can be transferred from one modality to another. Following the demonstration by Konkle, Wang, Hayward, and Moore (2009) that visual motion adaptation can produce a tactile motion aftereffect, we have conducted a series of experiments investigating whether visual adaptation to orientation can also affect our sense of touch. In each experiment a pair of points, separated by 30 mm, was touched on the subject’s forehead, the task being to determine whether an imaginary line between the points tilted clockwise or anticlockwise from vertical. In Experiment 1, subjects were adapted to visually presented tilted gratings (15°) and the effect on two-point orientation discrimination determined. Results (*n* = 13) indicate that visual tilt adaptation produces a shift in the perceived tactile tilt in an analogous fashion to the visual tilt aftereffect. The magnitude of the effect was approximately 2.5°. In Experiment 2, we investigated the reference frame of the aftereffect. Subjects repeated Experiment 1 but with their heads tilted during the adaptation phase by 15° so that the gratings were aligned with the vertical meridian of the retina. The resulting tactile aftereffect (*n* = 14) was approximately 2.6°, similar to the effect in Experiment 1. This suggests that the effect is determined by a gravitational rather than a purely visual frame of reference. We are currently examining a condition in which subjects adapt to a vertical grating with heads tilted at 15°. This adaptation is known to produce a visual tilt aftereffect; however, if the effect described here is gravitationally based, this adaptation should produce no tactile illusion.

## References

[bibr57-2041669515614148] KonkleT.WangQ.HaywardV.MooreC. I. (2009) Motion aftereffects transfer between touch and vision. Current Biology 19: 745–750.1936199610.1016/j.cub.2009.03.035PMC3398123

# Sub article: 4.1 How Attention Priming Dominates Perception: Influences on Attentional Choice, Crowding, Masking and Liking of Stimuli

**Authors:** Árni Kristjánsson

**Affiliations:** University of Iceland, Iceland

## Abstract

Priming of attention has often been studied as if it is a curiosity in visual search. I propose that its’ dominating influence on perception yields fundamental insights into perceptual processing, revealing how the visual system manages to keep the visual world stable and update key representations quickly and efficiently. I will describe recent results from four rather different experimental approaches that highlight the strong influence that attention priming plays in various aspects. These studies show that attentional priming has a strong effect on various measures apart from its influence on visual search: (a) Priming almost completely determines free choice between two different stimuli; (b) Priming can release items from visual crowding; (c) Priming can aid recovery from masking effects; (d) Priming influences the evaluation of stimuli, in other words how much they are liked. All these results show how the visual system strongly prefers items that were previously attended, and more broadly showing how the past determines the present in visual perception.

# Sub article: 4.2 Rapid Priming and Adaptation Modulate Prediction-of-Motion: Implications for the Involved Levels of Processing

**Authors:** Gianluca Campana, Luca Battaglini

**Affiliations:** University of Padova, Italy

## Abstract

Prediction-of-Motion can be investigated with a time-to-contact (TTC) estimation of a target passing behind an occluder. Previous findings have shown that TTC estimation can be modulated by motion adaptation of the occluded area, suggesting that the mechanism underlying Prediction-of-Motion could be a perceptual one. Here, we exploited rapid forms of visual motion priming and adaptation that were shown to occur at the earliest levels of visual cortical processing. In particular, while TMS studies found an involvement of early and intermediate visual areas along the cortical hierarchy in the rapid motion aftereffect, psychophysical studies found that rapid visual motion priming could not be elicited by stimuli (such as complex motion) processed by intermediate or higher level visual areas. By using different exposure times of directional motion (the same producing either rapid visual motion priming or rapid motion aftereffect) presented in the occluder area just before the target disappeared behind it, we found that longer (yet sub-second) exposures to motion in the same direction of the target increased TTC estimates, whereas shorter exposures decreased TTC estimates. These results indicate that rapid forms of motion adaptation and motion priming modulate extrapolated motion. Thus, motion extrapolation might occur at the first levels of cortical motion processing, where these rapid mechanisms take place.

# Sub article: 4.3 Visual Adaptation to Changes in Statistical Properties of the Environment

**Authors:** Pascal Mamassian, Kyle C. McDermott

**Affiliations:** CNRS & Ecole Normale Supérieure, Paris, France

## Abstract

Repeated stimulus presentations can lead to dramatic changes in the perception of subsequent stimuli. Two such phenomena are biased perception (aftereffects) due to adaptation and increased sensitivity due to perceptual learning. Here, we study the interaction of these two phenomena.

Observers were asked to indicate whether the direction of motion of a briefly presented field of random dots was to the left or right of a cued direction. Stimuli were presented by method of constant stimuli with directions of motion chosen from a nonuniform distribution designed to induce a bias. This was achieved by manipulating the frequency with which different directions of motion were presented: For both the left and right halves of the stimulus distribution, the left end of the range was shown few times and the right end of the range was shown many times. Observers trained on this task for an hour a day for five consecutive days.

The data demonstrate both a bias in perceived direction of motion due to adaptation and a reduction in discrimination threshold due to learning. Both phenomena asymptotically approach some maximum magnitude both within and across days with some of the effects carrying over from one day to the next. These results are consistent with a model based on a process where stimuli induce slight changes in the tuning of neural populations coding for motion direction. Such alterations of these tuning functions are in line with physiological measurements and similar models have been used to explain various phenomena associated with long-term exposure to the image statistics of natural scenes.

# Sub article: >5.1 Integrating Visual Information Overtime

**Authors:** Geoffrey M. Boynton, H.- Wallander Bjorn

**Affiliations:** University of Washington, USA

## Abstract

Perceptual decisions often require integration of information across time. Even a static scene is effectively sampled overtime via saccades. We are interested in how humans combine serially presented information into overall judgments of simple visual stimuli.

For the first experiment, subjects viewed a sequence of 10 discs presented sequentially varying in both spatial position and size. Subjects were precued on each trial to indicate either the perceived center or the mean size of the discs. A set of 10 weights was obtained for each subject that reflects the relative influence that discs in each temporal interval had on the overall judgment of position or size.

For estimating the central location, subjects show a much stronger weight for early discs—a “primacy” effect. For mean size, however, subjects showed stronger weighting for discs presented later in the sequence—a “recency” effect.

In subsequent experiments, subjects were asked to indicate the mean direction of motion of a sequence of coherently moving dots and the mean facial expression of a sequence of faces ranging from happy to sad. Both of these experiments showed a recency effect, with the latest directions of motion and facial expressions contributing much more weight than earlier stimuli.

Together, these experiments show that the way subjects integrate information across time to make a decision about central tendency depends on the stimulus domain. We discuss two explanations for this. First, it may be that these differences reflect different neural mechanisms involved with bottom-up mechanisms that integrate information overtime. Second, these differences may reflect different high-level processes that have different decision-making constraints. For example, it may be optimal to make early, rapid decisions about mean spatial location in order to make quick eye movements to the perceived center. Decisions about other stimulus domains like facial expression might be optimally made by emphasizing later information that better reflect the current state of a changing stimulus.

# Sub article: 5.2 Factors That Influence Judging and Guessing About Probabilistic Event Sequences

**Authors:** József Fiser

**Affiliations:** Brandeis University, MA, USA

## Abstract

Previous studies have reported several factors, including prior knowledge, past experience, immediately preceding events, and rate of event repetitions that influence humans’ ability to predict and perceive sequentially occurring probabilistic events. However, many of these factors are correlated and most earlier studies made little effort to disentangle their confounding effects. I will present a series of human behavioral experiments, in which we systematically inspected the separate and joint effects of these factors within a simple visual perceptual paradigm. We found that, rather than simply balancing past and present statistics, the best model describing human performance is probabilistic and it assumes a parallel working of several factors: (a) reliance on prior statistical knowledge of the sequence as a function of stimulus uncertainty, (b) a “regression to the mean” kind of effect that could reflect a general strategy of noncommitment, and (c) an independent short-term repetition effect which influences performance asymmetrically.

# Sub article: 5.3 You Know You Can’t Do That: Effects of Experience and Abruptly Imposed Movement Constraints on Sequential Estimation of Success Probability

**Authors:** Holly E. Gerhard, Laurence T. Maloney

**Affiliations:** Stanford University, CA, USA; New York University, USA

## Abstract

In typical sequential judgment tasks, the subject is told the probability of occurrence of one of two possible mutually exclusive outcomes in advance. In many everyday tasks, however, we must somehow estimate the probability of the various outcomes. One possible source of information is our actual success or failure in repeated trials. Another possibility is that we base our estimate in part on a model of how our body works. The evident advantage of the latter is that it can be much less painful than trial and error learning. The evident disadvantage is that reliance on incorrect models can lead to errors in estimating our own capabilities.

In two experiments, we examined human estimation of the probability of success in a repeated motor task. Subjects performed 100 speeded pointing trials on a horizontal touch screen. The target was a bulls-eye with a small center circle and a larger surround. Before each trial, subjects estimated their probability of hitting the target and their probability of hitting the center circle. They then attempted to hit the target. After every trial they received feedback. We compared human performance to an ideal Bayesian updating model for the first 40 trials. Performance was consistent with Bayesian updating and the gradual accumulation of information in the form of a beta distribution prior.

However, without warning, during trials 41–70, we either fixed their elbow at 90° (Experiment 1) or constrained them to use their nondominant hand (Experiment 2). This intervention mimics the consequences of unexpected loss of motor function due to injury or stroke. At the 71st trial, we removed the constraint.

We found that—for many subjects—added constraints led to large and persistent underestimation of target-center hit rates even after normal movement was restored. Subjects based their estimates not only on their experience of success and failure but also on an incorrect model of how constraint would affect their performance.

Support: NIH NEI EY019889 and the Alexander v. Humboldt Foundation.

Note: These abstracts have not been edited and are printed as submitted.
